# Convergent validity of commonly used questions assessing physical activity and sedentary time in Swedish patients after myocardial infarction

**DOI:** 10.1186/s13102-022-00509-y

**Published:** 2022-06-24

**Authors:** Amanda Lönn, Lena Viktoria Kallings, Mats Börjesson, Örjan Ekblom, Mattias Ekström

**Affiliations:** 1 Department of Physical Activity and Health, The Swedish School of Sport and Health Sciences, Gymnastik- Och Idrottshögskolan (GIH), Lidingövägen 1, 114 33 Stockholm, Sweden; 2 Women’s Health and Allied Health Professionals Theme Medical Unit Occupational Therapy and Physiotherapy, 171 76 Stockholm, Sweden; 3 Unit of Family Medicine, Department of Public Health and Caring Sciences, Uppsala University, 751 05 Uppsala, Sweden; 4 Center for Health and Performance, Department of Food, Nutrition and Sport Science, University of Gothenburg, Gothenburg, Sweden; 5 Department of Neuroscience and Physiology, Sahlgrenska Academy & Sahlgrenska University Hospital, 411 24 Gothenburg, Sweden; 6 Division of Cardiovascular Medicine, Department of Clinical Sciences, Danderyd Hospital, 182 88 Stockholm, Sweden

**Keywords:** Questionnaire, Accelerometer, Coronary heart disease, Physical activity, Validation, Sedentary time

## Abstract

**Background:**

Guidelines recommend regular physical activity (PA) and decreased sedentary time (SED) for patients after myocardial infarction (MI). Therefore, valid self-assessment of PA is vital in clinical practice. The purpose of this study was to assess the convergent validity of commonly used PA and SED questions recommended by the National Board of Health and welfare (NBHW) and national SWEDEHEART-registry using accelerometers as the reference method in patients after MI.

**Methods:**

Data were obtained 2017–2021 among Swedish men and women (180 assessments). Participants answered five commonly used PA and SED-questions (by NBHW and SWEDEHEART) and wore an accelerometer (Actigraph GT3X) for seven days. Convergent validity was assessed gradually by; Kruskall Wallis-, Sperman rho, Weighted Kappa- and ROC-analyses. Misclassification was explored by Chi-square analyses with Benjamini–Hochberg adjustment.

**Results:**

The strongest correlation (r = 0.37) was found for the SED-GIH question (NBHW). For PA, no specific question stood out, with correlations of r = 0.31 (NBWH), and r = 0.24–0.30 (SWEDEHEART). For all questions (NBHW and SWEDEHEART), there was a high degree of misclassification (congruency 12–30%) affecting the agreement (0.09–0.32) between self-report and accelerometer assessed time. The SED-GIH, PA-index and SWEDEHEART-VPA had the strongest sensitivity for identifying individuals with high SED (0.72) or low PA (0.77 and 0.75).

**Conclusion:**

The studied PA and SED questions may provide an indication of PA and SED level among patients with MI in clinical practice and could be used to form a basis for further dialogue and assessment. Further development is needed, since practical assessment tools of PA and SED are desirable.

**Supplementary Information:**

The online version contains supplementary material available at 10.1186/s13102-022-00509-y.

## Introduction

Cardiovascular disease (CVD) is the major cause of death in Europe [1]. There is evidence that regular physical activity (PA), including exercise and everyday PA as well as limited sedentary time (SED) lower the risk of hospitalization and premature mortality [2–5].

International guidelines strongly recommend individualised PA and SED recommendations for patients with CVD [6, 7]. Therefore, valid self-reported assessment is a vital component in clinical practice. Questionnaires are easy to use and an inexpensive way of describing previous PA behaviour, type of activity and the relative intensity [8]. However, questionnaires suffer from potential errors due to social desirability and the difficulty to accurately estimate duration and absolute intensity [8, 9].

“The Swedish National Board of Health and Welfare” (NBHW) recommends two PA questions (NBHW-PA questions) to assess levels of everyday PA, exercise, and a combination of these [10]. They also recommend a question regarding the amount of time spent sedentary (SED-GIH) [11]. A previous study explored the association of the NBHW-PA and SED questions with health care utilisation and mortality among patients with CVD [3]. The results indicated that higher levels of everyday PA and lower time spent sedentary were associated with lower readmission rates. In addition, both higher exercise frequency and everyday PA were linked to a lower mortality risk [3]. The Swedish national quality registry for acute coronary and cardiac rehabilitation care, “SWEDEHEART”, contains questions based on PA-recommendations by Haskell et al. [12], focusing on moderate and/or vigorous intensities of PA (SWEDEHEART-PA questions) [13]. Data based on the SWEDEHEART-questions have linked high levels of both moderate and vigorous PA with a lower risk of rehospitalization and risk of all-cause mortality in patients with (myocardial infarction) MI [2].

Thus, the current questions seem to have an acceptable predictive validity for identifying individuals with a higher risk of rehospitalization and all-cause mortality [2, 3]. However, there is still a gap in knowledge of the convergent validity of the questions commonly used in clinical practice among individuals with atherosclerotic CVD i.e., can the questions be used to estimate individuals PA level. The objective of the present study is to assess the convergent validity of the PA and SED questions in patients after a MI using accelerometers as the reference method.

## Methods

This was a single center study performed at the Department of Cardiology at Danderyd’s hospital, Stockholm, Sweden. Individuals were informed of the study and invited to participate by a nurse at the first routine follow-up visit, six to eight weeks after MI.

Data were collected via questionnaires, accelerometers, diaries, and the national quality registry SWEDEHEART between October 2017 and May 2021. The participants answered a questionnaire concerning PA and SED both at six to eight weeks and at 10–12 months post MI. Immediately after the visits, an accelerometer with a diary were posted to the participant, with instructions on how to wear the device and to start the accelerometer assessment directly. After the measuring period, participants returned the accelerometer and the diary by prepaid postage to the Swedish School of Sport and Health Science. Covariates were collected from a sub-register of SWEDEHEART, the SEPHIA-registry, focusing on outpatient cardiac rehabilitation up to one year after MI from the responsible cardiac department [14]. The following variables were obtained: gender, age, occupation, smoking habits, body mass index (BMI), systolic and diastolic blood pressure, left ventricular ejection fraction (LVEF), diabetes (ICD E.10-E.11) and smoking habits.

### Study participants

Individuals aged 18–80 years with newly diagnosed MI (ICD I21) registered in the SWEDEHEART registry were included in the study. Individuals who were physically disabled (wheelchair dependent) or with reduced ability to answer the questions were excluded by not being asked for participation.

### Physical activity and sedentary time questionnaire

Six commonly used PA and SED questions were included in the study (the NBHW-PA, SED-GIH questions and the SWEDEHEART-PA questions).

The NBHW-PA and SED questions use different time frames. The NBHW-PA questions focus on PA in an ordinary week while the SED question focuses on an ordinary day.

### NBHW-PA questions [10]

- Everyday PA: “During a regular week, how much time (in minutes) are you physically active in ways that are not exercise, for example walks, bicycling, or gardening? Add together all activities lasting at least 10 min”. Seven fixed answers were available; “no time”, ” < 30”, “30–60”, “60–90”, “90–150”, “150–300” and “ > 300”.
- Physical exercise: During a regular week, how much time do you spend exercising on a level that makes you out of breath, for example running, fitness class, or ball games? The questions had six fixed answers; “no time”, “ < 30”, “30–60”, “60–90”, “90–120” and “ > 120”.

The questions of everyday PA and physical exercise formed a validated PA-index (3–19 points) of total PA level [10]. This was obtained by multiplying the category of exercise (one to six) by two, to account for a potentially higher intensity and then adding the category of everyday PA (one to seven). The same index has been used in previous surveys to assess the approximate number of individuals who achieve ≥ 150 min of MVPA [10]. The cut-off was set at nine.

### NBHW SED-GIH question [11]

- Sedentary time: “How much time (in hours) do you sit during a normal day, excluding sleep?” [11]. There were seven answer options; “Virtually all day”, “13–15”, “10–12”, “7–9”, “4–6”, “1–3” and “Never”.

### SWEDEHEART-PA questions

The PA questions in the SWEDEHART registry focus on PA during the previous week and are listed below. The individuals can choose between 0–7 days/week [15]

- SWEDEHEART-MVPA: “Number of physical activity sessions of at least 30 min (two 15-min sessions can be combined into one 30-min session) in the last 7 days, with a minimal intensity of fast walking”.

The following two questions separate moderate and vigorous intensity levels of PA.

- SWEDEHEART-MPA*:* How many days in the last week did you do at least 30 min, total time (at least 10 min at a time) of physical activity that made you slightly out of breath and gave slightly elevated heart rate?
- SWEDEHEART-VPA: How many days during the last week did you do some form of continuous vigorous physical activity /exercise, (at least 20 min), that made you out of breath and gave you elevated heart rate?

### Accelerometer data

An accelerometer provides information on body movements, which can be translated to data on PA, using validation algorithms. Such translation gives reasonably valid data of duration, frequency, and absolute intensity of PA and SED [16, 17]. Accelerometers (Actigraph GT3X monitor, Pensacola, Florida, USA) were used as the convergent PA and SED assessment method. Participants were encouraged to wear the monitors on the right hip, 24 h for ten consecutive days [18], except in water-based activities. The participants used a diary during the period they carried the accelerometer. They noted the time they went to bed and woke up as well as time without the accelerometer. These periods (if ≥ 40 min) were excluded from the accelerometer analyses. When diary data of sleeping hours were missing, the time between 10:00 PM and 07:00 AM was considered night and excluded from the accelerometer analyses.

The accelerometers and data files were processed using the software Actilife, version 6.13.4 (ActiGraph llC, Pensacola, Fl, USA). Data were collected tri-axially using a sampling rate of 30 Hz and, after extraction, data were down-sampled and saved as 60 s epochs, with activity intensity based on vector magnitude. Normal frequency filter was applied. Additionally, the Choi algorithm was used for validate non-wear time [19], defined as a minimum of 90 consecutive minutes with no movement, i.e., 0 counts per minute (cpm), with an allowance for a maximum amount of movement of two minutes with intensities up to 199 cpm.

Cut-points were used to describe the daily PA behaviour, using the following components: time spent in sedentary (SED, 0–199 cpm), light physical activity (LIPA, 200–2689 cpm), moderate PA (MPA 2690–6166) and vigorous physical activity (VPA, ≥ 6167 cpm) [20, 21]. The mean daily time in SED, LIPA, MPA, and VPA was calculated as the sum of each variable on all valid days divided by the number of valid days. In addition, time in MPA and VPA was summed up and termed moderate-to-vigorous PA (MVPA).

### Statistics

To be included in the analyses, individuals had to answer the questionnaire and have valid accelerometer data for at least seven days defined as ≥ 600 min of data per day after non-wear time had been excluded.

The descriptive data are presented as median and 25th and 75th percentiles (Q1–Q3), or number and proportions. Before Spearman and weighted Kappa analysis, the continuous accelerometer data were divided into the same categories as the different answer options for PA (everyday PA, exercise, number of days in MVPA, MPA, VPA) and SED questions. For the PA-index, the average number of minutes in MVPA was kept as a continuous variable.

Convergent validity was explored gradually:

Kruskal–Wallis analyses with Bonferroni correction were performed to investigate the differences in median accelerometer derived time between the different categories of the PA and SED questions.

- Multiple linear regressions were performed to control for repeated measurements among individuals with two assessments. The study-id was used as a covariate.
- Correlations between the categorised accelerometer data and the PA and SED-GIH questions were calculated using Spearman’s rho (r). The associations were interpreted as weak (r < 0.10), modest (r 0.1–0.3), moderate (r 0.3–0.5), strong (r 0.5–0.8) or very strong (r 0.8–1.0) [22]. To explore differences in correlations, r-to-z transformations were performed.
- Weighted kappa analyses were conducted to assess the agreement between PA and SED-GIH questions with the categorized accelerometer data (n.a. for the PA-index). The agreement (Kappa) was interpreted as poor (< 0.20), fair (0.21–0.40), moderate (0.41–0.60), substantial (0.61–0.80) or almost perfect (0.81–1.00) [23].
- Receiver operating characteristics (ROC) curves were calculated. The answer options were kept as ordinal variables and the accelerometer data were used as state variable (golden standard) and dichotomised at a cut-off value. The cut-off for achieving the PA recommendations was ≥ 150 min a week of MPA for the everyday PA question, ≥ 75 min a week of VPA for the exercise question and ≥ 150 min of MVPA for the PA-index (NBHW-PA questions). For the SWEDEHEART questions the accelerometer cut-off was set at ≥ 20 min of total VPA for at least 3 days a week and ≥ 30 min of total MPA or MVPA for at least 5 days a week. Individuals with ≥ 9.5 h of SED per day were categorized as high SED, while individuals with less time spent sedentary were categorized as low SED. This cut-off was chosen based on international PA [6, 7] and SED [24] recommendations and risk assessments. For the ROC analyses, data are presented as the area under the curve (AUC) and 95% confidence intervals. AUC ranges from 0–1, where a poor model has an AUC value of 0.5 and an excellent model has a value of 1. Sensitivity and specificity analyses were used to identify the proportion of true positive and true negative answers to the PA and SED questions, based on the dichotomized accelerometer data. The point estimate (answer alternative) that generated the strongest combination of sensitivity and specificity was chosen.

Lastly, calculations in Excel were performed to assess how well the PA and SED-GIH questions corresponded to the accelerometer derived data, either congruent, or over- and underestimating. Then, Chi square analyses with Benjamini–Hochberg adjustments were used to assess differences in congruence, under-, and overestimation between gender, age, LVEF, systolic blood pressure, diabetes, and BMI. All statistical analyses were performed using SPSS 27.0 software (IBM Corp., Armonk, NY, USA).

## Results

A total of 123 individuals answered the questionnaire and provided complete accelerometer data were included (Table 1). The median age was 67 years, with a majority of men with a preserved LVEF. A high proportion of the participants was regularly physically active. The accelerometer data showed 77% achieved at least 150 min of MVPA during a week. However, 47% had ≥ 9.5 h of daily SED. Individuals (n = 65) excluded from the analyses did not significant differ from included in gender but were significant younger median 58 (IQR 16) years. Of included individuals, 56 individuals also provided data from a second assessment (10–12 months after MI), leading to 179 complete assessments included in the analyses. There was an internal drop-out for the NBHW, SED (*n* = 2), (*n* = 3) everyday-PA and exercise (*n* = 2) questions.

**Table 1:** Baseline characteristics of participants (n = 123)^a^

Gender, men	98 (80%)
Age, years	67 (59–72)
*Employment*	
Working	39 (32%)
Sick leave	6 (5%)
Retiree	73 (59%)
Other	5 (4%)
BMI^b^, kg/m^2^ (n = 122)	26 (24–29)
Diabetes (n = 122)	20 (16%)
Systolic blood pressure, mmHg	123 (112–130)
Diastolic blood pressure, mmHg	73 (67–80)
*LVEF*^*c*^	
≥ 50%	92 (75%)
40–49%	26 (21%)
< 40%	5 (4%)
*Smoking*	
Never smoker	61 (50%)
Previous smoker	55 (45%)
Smoker	7 (6%)
Daily wear time in minutes	912 (862–946)
Daily minutes in MVPA^d^	47^f^ (24–79)
Daily minutes in SED^e^	558^f^ (500–614)

^a^Data presented as median (25–75 percentile) or as number and percent

^b^Body mass index

^c^Left ventricular ejection fraction

^d^Moderate and vigorous physical activity

^e^Sedentary time

^f^Assessed by accelerometer

### Convergent validity

**Fig. 1:**
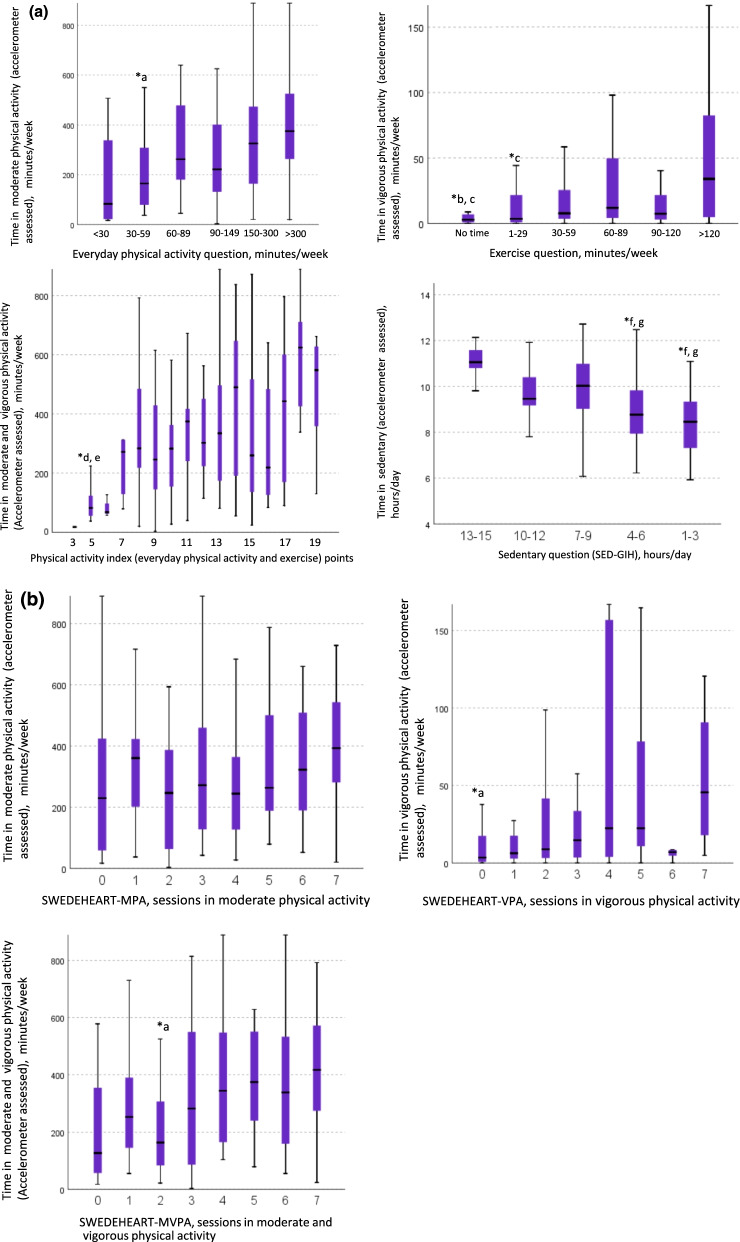
**a** Accelerometer assessment of physical activity and sedentary time across categories of the NBHW-questions (n = 176). *Differed from (*p* < 0.05); ^a^> 300 min, ^b^60–89 min, ^c^> 120 min. ^d^18 points, ^e^19 points, ^f^10–12 h, ^g^13–15 h. **b** Accelerometer assessment of physical activity across categories of the *SWEDEHEART*-questions (n = 179). *Differed from (*p* < 0.05); ^a^7 sessions

In the Kruskall-Wallis analyses we noticed differences (p < 0.05) in accelerometer collected time for different categories of the NBHW-PA and SED questions (Fig. 1a). For everyday PA, a difference was found in MPA between the categories “30–59” and “ > 300” minutes. Regarding the exercise question, we found differences in VPA between “no time” compared to “60–89” and “ < 120” minutes respectively, as well as a difference between the categories “1–29” and “ > 120″ minutes. The PA-index could detect differences in accelerometer derived time in MVPA between individuals categorised as 5 vs 18 or 19. For SED, there were differences in accelerometer assessed time for individuals self-reporting the two lowest categories compared to individuals reporting > 10 h. For the SWEDEHEART-PA questions there were significant differences in accelerometer assessed time for the SWEDEHEART-VPA and MVPA questions (Fig. 1b). For the SWEDEHEART-VPA question, differences in time in VPA were seen between individuals categorised to zero and seven sessions. For MVPA the differences in time of MVPA were seen between category two and seven.

In the multiple linear regression (Additional file 1), the study-id did was not a significant predictor for the relationships, there for all assessments (n = 179) were included in the analyses.

The correlation between the SED question and categorical accelerometer data was moderate (r = 0.37), but the agreement was weak (0.09). However, the ability to classify individuals sitting ≥ 9.5 h was good, with an acceptable level of both sensitivity (72%) and specificity (62%) (Table 2).

**Table 2:** Convergent validity for self-reported estimates of physical activity and sedentary using accelerometer assessed time as reference

Question	Correlation ^a^	Agreement ^b^ (95% CI)	Area under the curve ^c^ (95% CI)	Sensitivity	Specificity
*NBHW-PA questions*					
Sedentary time (n = 177)	0.37	0.09 (0.05–0.12)	0.69 (0.60–0.77)^d^	0.72	0.62
Everyday physical activity (n = 176)	0.31	0.31 (0.16–0.46)	0.68 (0.59–0.78)^e^	0.65	0.62
Exercise (n = 177)	0.31	0.18 (0.09–0.27)	0.69 (0.57–0.82)^f^	0.47	0.85
Physical activity index (n = 176)	0.31	n.a	0.65 (0.54–0.76)^g^	0.77	0.50
*SWEDEHEART-PA questions (n* = *179)*					
SWEDEHEART-MVPA^k^	0.30	0.32 (0.18–0.46)	0.64 (0.56–0.72)^h^	0.66	0.55
SWEDEHEART-MPA^l^	0.28	0.19 (0.07–0.32)	0.62 (0.53–0.71)^i^	0.53	0.72
SWEDEHEART-VPA^m^	0.24	0.10 (0.02–0.18)	0.61 (0.50–0.72)^j^	0.75	0.48

^a^Spearman rho

^b^Weighted Kappa

^c^ROC-analyses

^d^Cut-off ≥ 9.5 h per day of SED

^e^Cut-off ≥ 150 min a week of MPA

^f^Cut-off ≥ 75 min a week of VPA

^g^Cut-off ≥ 150 min of MVPA

^h^≥ 5 sessions a week of MVPA

^i^Cut-off ≥ 5 sessions a week of MVPA

^j^Cut-off ≥ 3 sessions a week of VPA

^k^Moderate and vigorous intensity physical activity

^l^Moderate intensity physical activity

^m^Vigorous intensity physical activity

For PA, the correlations between the PA questions and categorical accelerometer data were modest to moderate (r = 0.24–0.31) and no specific question had a significantly stronger correlation (p > 0.47). In general, the weighted kappa analyses showed a poor to fair agreement between the answer categories and categorised accelerometer data (agreement 0.10–0.32) with the strongest agreement (fair) for the SWEDEHART-MVPA and the NBHW everyday PA questions. Results from the ROC analyses are presented in Table 2, and graphical results in Additional file 2. To classify individuals as achieving ≥ 150 min of MVPA the NBHW PA-index had the best sensitivity (77%). Meanwhile the NBHW exercise question had the best specificity (85%) for identifying individuals not fulfilling ≥ 75 min a week of VPA (Table 2). Correlation, agreement, and area under the curve for the two specific timepoints are presented in Additional file 3, showing random but not systematic differences.

### Over- and under-reporting

**Fig. 2:**
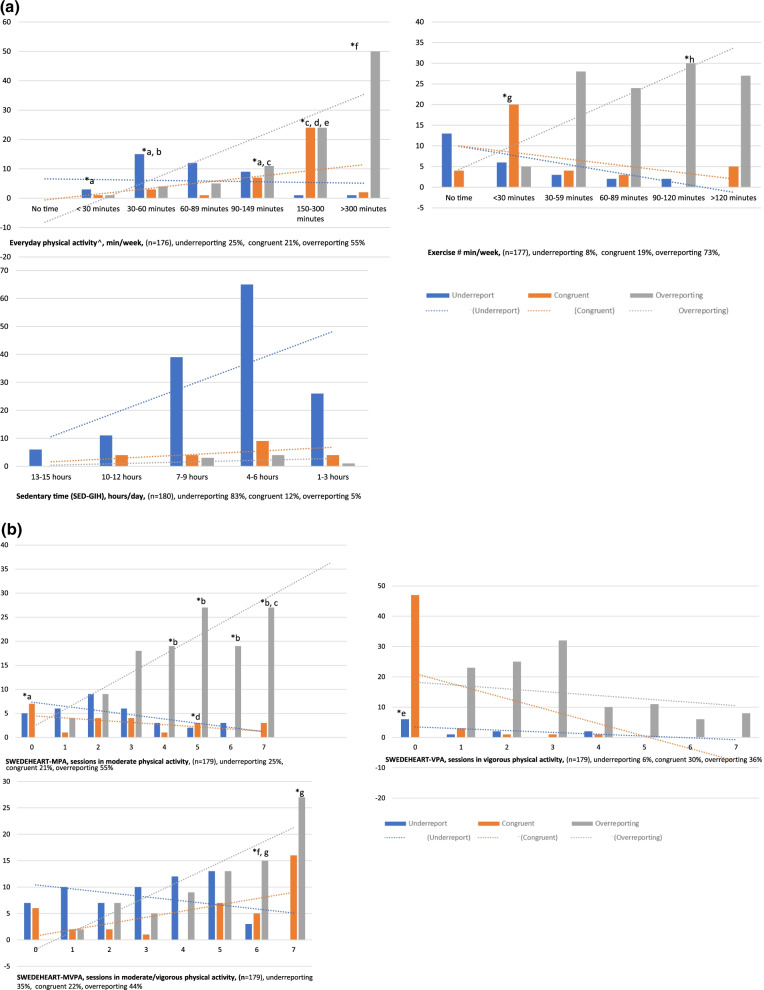
**a** Congruence and misclassification in the *NBHW-*questions compared to accelerometer assessed time in physical activity and sedentary. ^Accelerometer data include physical activity at a moderate intensity for the same duration as the everyday PA question different answer categories. ^#^Accelerometer data include physical activity at a vigorous intensity for the same duration as the exercise question different answer categories. *Differed from (*p* < 0.05); ^a^under report compared to 150–300 min and 300 min, ^b^under report more than 90–149 min, ^c^more congruent than > 300 min, ^d^more congruent than 30–60 min and 60–89 min, ^e^over report more than 30–60 min, ^f^over report more than all other categories. **b** Congruence and misclassification in the *SWEDEHEART-*questions compared to accelerometer assessed time in physical activity. *Differed from (*p* < 0.05); SWEDEHEART-VPA; ^a^ more congruent compared to 3–7 sessions, ^b^overreport more compared to 1–2 sessions, ^c^overreport more compared to 3 sessions, ^d^overreport more compared to 0–2 sessions, SWEDEHEART-VPA ^e^more congruent compared to 1–4 sessions; SWEDEHEART-MVPA, ^f^underreport more compared to 0, 1, 3 and 4 sessions; ^g^overreport more compared to 1 session

Congruence between individuals’ self-reported PA and SED levels compared to the accelerometer measured data varied between 12 and 30% (Fig. 2a, b). In general, participants with a high self-rated PA level over-reported the PA to a larger degree. For SED, the majority (83%) under-reported their sedentary time; however, there were no differences in misclassification between the different SED categories.

There were few other factors affecting the reporting pattern. Older individuals over-reported everyday PA to a higher degree than younger (63% vs 44%). Men under-reported the number of PA sessions in MVPA (SWEDEHEART) compared to women (39% vs 18%) (Additional file 4). Lastly, older and overweight/obese individuals over-reported time in VPA (SWEDEHEART), compared to younger individuals (68% vs 60%) and those with a BMI < 25 kg/m^2^ (71% vs 53%).

## Discussion

The main finding of this study was, that for the PA and SED questions (by NBHW and SWEDEHEART) frequently used in clinical practice, there was a high degree of misclassification (over- and underreporting), with modest to moderate correlation and poor to fair agreement. Nonetheless, they may provide some broadly indication about PA and SED level, which could form the basis for further discussion and assessment on an individual level in clinical practice.

### Sedentary question (SED-GIH)

The updated European guidelines of CVD prevention highlight the importance of decreasing SED; therefore it is important that the convergent validity of the SED-GIH question has an acceptable level. The correlation to accelerometer assessed SED (r = 0.37) was somewhat lower compared to a study in a general population that showed a correlation between the SED-GIH question and total stationary time (r = 0.48) [11]. Convergent validity for other SED questionnaires among individuals within cardiac rehabilitation has previously been explored in two small studies, where modest (r = 0.19–0.21) correlations to accelerometer collected data [25, 26] were found.

The agreement between the SED-GIH question and SED time assessed by accelerometer was weak. This might be due to eight out of ten participants underestimating their sedentary time causing misclassification. The difficulty to estimate SED has been shown in other studies, with misclassifications of minus 85 min per day [27] and under-estimations of SED of approximately 70% [11]. To decrease the risk of misclassification, Gardner et al. suggested asking about time spend in seated activities, e.g. time of tv-viewing or computer use [28] instead of questions about sitting time. Despite the under-estimation of sitting time, ROC-analyses showed a modest ability (0.69) to identify individuals with more than 9.5 h/day of accelerometer assessed SED [24]. This is supported by Kallings et al. in a general population study (0.71) [11].

### The NBHW and SWEDEHEART-PA questions

For PA, the correlation for the NBHW and SWEDEHEART questions with accelerometer derived data was modest to moderate. There were no significant differences in correlation for the NBHW-PA questions compared to the SWEDEHEART ones. The correlation of NBHW PA-index with accelerometers as a criterion method showed similar results to a study in a general population, r = 0.27 (vs 0.31) for MVPA min/day [10]. To our knowledge, few studies explore convergent validity between self-rated and accelerometer assessed PA for individuals with CVD. However, Biswas et al. found a moderate correlation (r = 0.49) between accelerometer assessed and self-rated time of MVPA in this group [26].

For the PA questions, agreement with time assessed by accelerometer was poor to fair (0.10–0.32) with the strongest agreement for the SWEDEHEART-MVPA and the NBHW everyday PA question. As with a previous study [27], there was a high degree of overestimation for all PA questions. Interestingly, there were small differences in misclassifications between groups, with women, obese and older participants over-reporting time and intensity of PA to a higher degree than men, individuals with a lower BMI and younger participants. This might be due to accelerometers not considering the individual’s physical capacity (relative intensity), unlike self-reported data. This is clinically important, as relative intensity is valuable for recommendations of PA based on the individual.

ROC-curve analysis of the PA-index was in line with a study in a general population (0.66) [10]. Thereto the PA-index had the strongest sensitivity (0.77) to identify individuals not achieving 150 min of MVPA collected by accelerometer, which indicates that the PA-index might be used to indicate low PA among patients with MI as well as in the general population. To identify individuals not achieving 3 times per week of VPA (accelerometer collected), the sensitivity was equivalent (0.75) for the SWEDEHEART-VPA question.

### Strengths and limitations

The most important strength of this study is that it includes a large cohort of CVD patients, focusing on individuals with MI. It also includes a higher proportion of older individuals compared to previous studies [10, 11]. Generalization of the results is possible as the median age and proportion of women is similar to patients included in the SEPHIA-registry [15]. However, included individuals were older than excluded, which may slightly affect the generalisability of the SWEDEHART-VPA and everyday-PA question, when older individuals were classified as overreporting ta a higher degree.

A limitation is the lack of consensus for cut-off values of accelerometer data for individuals with CVD. This is troublesome and the time spent at different levels of PA intensity should therefore be interpreted with caution, especially in patients with reduced cardiorespiratory fitness, which leads to differences in absolute and relative intensity [18]. In this study, where a majority had a preserved LVEF, we used cut-off points in line with several previous studies on general populations [27] and patients in cardiac rehabilitation [18].

Accelerometers were used as the criterion method. However, accelerometer measurements do have disadvantages. For example, information about relative intensity and activities such as bicycling, strength training, and swimming, all common types of exercise within cardiac rehabilitation [6], are omitted. This might have led to the differences in self-rated and accelerometer assessed PA levels. In addition, the accelerometer assesses stationary behaviour and not sitting time per se, which the SED-GIH question focuses on. This might have contributed to the high amount of under-reporting. The same limitation was apparent when comparing accelerometer collected MPA with the everyday PA question, focusing on activities performed in the everyday, not specifying the intensity.

Another limitation is that participants may have become more conscious about their PA behaviour during the study, affecting how they answered the questions and their PA behaviour during the measurement period. The participants answered the questionnaire prior to wearing the accelerometer, this may be a limitation when the SWEDEHEART-PA questions focus at PA the last week, however several studies indicate that accelerometer data collected over seven days use to be consistent between weeks (high reliability) [29–31]. Non-wear time is important to consider, since it affects PA and SED assessed by the accelerometer [32]. Thus, using a diary to register sleep and non-wear time attenuated this limitation. Before analyses, these times were excluded. Another strength is that all PA and SED questions consist of predetermined answer categories, which have been shown to increase validity compared to open answer questions [10].

Exploring an individual’s total PA behaviour is complex and these questions have a limited convergent validity and low, precision. Therefore, a combination of both accelerometer derived data and questionnaires could be recommended [8, 9].

## Conclusion

The present study is clinically important as it focuses on commonly used SED and PA questions to patients with CVD, where regular PA and low SED both have a central role in cardiac rehabilitation. The convergent validity of the SED and PA questions is poor to moderate compared to accelerometer assessed data. The SED-GIH question had the strongest correlation. However, for PA we could not identify any preferable question. In spite of the risk of misclassification when using questionnaires, the questions seem to be a practical and acceptable method that may provide some indication about PA-level, which could form the basis for further discussion and assessment on an individual level.

## Supplementary Information

**Additional file 1**. Correlations between self-rated PA level, SED and study-id with accelerometer assessed PA and SED time.

**Additional file 2**. Graphical results of the ROC analyses for the specificity and sensitivity of the PA and SED questions compared to accelerometer collected data.

**Additional file 3**. Convergent validity for self-reported estimates of physical activity and sedentary using accelerometer assessed time as reference for time point one (n=123) and two (n=56).

**Additional file 4**. Congruence between self-reported PA and SED compared to the accelerometer assessed time for different strata.

## Data Availability

The datasets generated and/or analysed during the current study are not publicly available due to ethical conditions but are available from the corresponding author on reasonable request.

## Abbreviations

AUC: Area Under the Curve
BMI: Body Mass Index
CVD: Cardiovascular disease
LVEF: Left Ventricular Ejection Fraction
MI: Myocardial infarction
MPA: Moderate physical activity
MVPA: Moderate and vigorous physical activity
NBHW: National Board of Health and Welfare
PA: Physical activity
ROC: Receiver operating characteristics
SED: Sedentary time
SEPHIA: The Secondary Prevention after Heart Intensive Care Admissions
SWEDEHEART: The Swedish Web-system for Enhancement and Development of Evidence-based care in Heart disease Evaluated According to Recommended Therapies
VPA: Vigorous physical activity
