# Supplementary material for: Convergent validity of commonly used questions assessing physical activity and sedentary time in Swedish patients after myocardial infarction
Source: BMC Sports Sci Med Rehabil. 2022 Jun 24;14:117. doi: 10.1186/s13102-022-00509-y (PMC9229098; doi:10.1186/s13102-022-00509-y)
Supplement: Supplementary file 2 — Additional file 2. Graphical results of the ROC analyses for the specificity and sensitivity of the PA and SED questions compared to accelerometer collected data. [file 13102_2022_509_MOESM2_ESM.pdf]

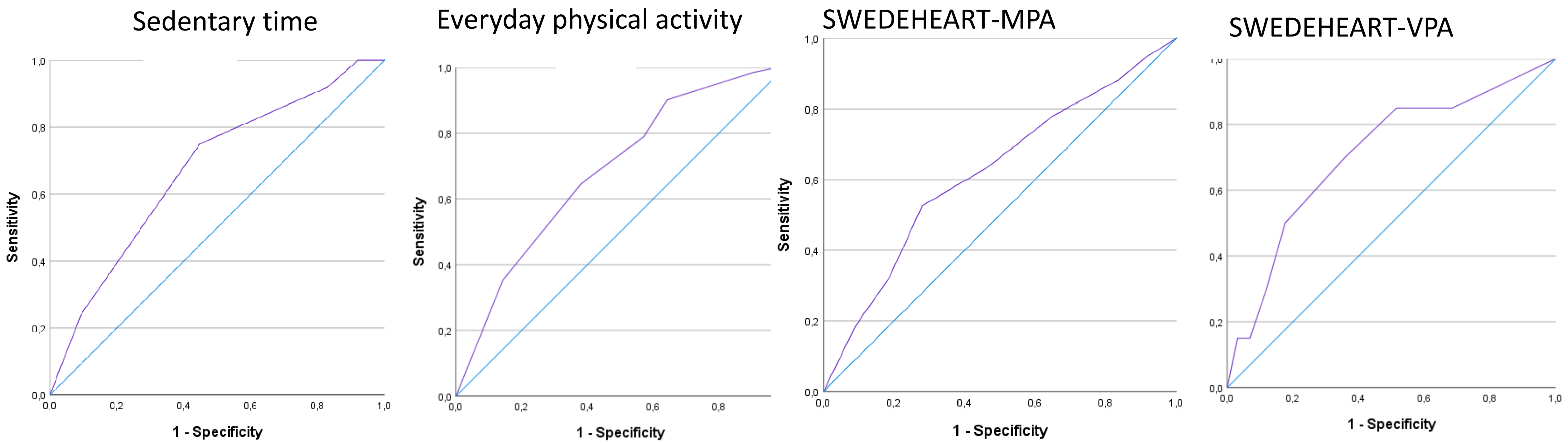

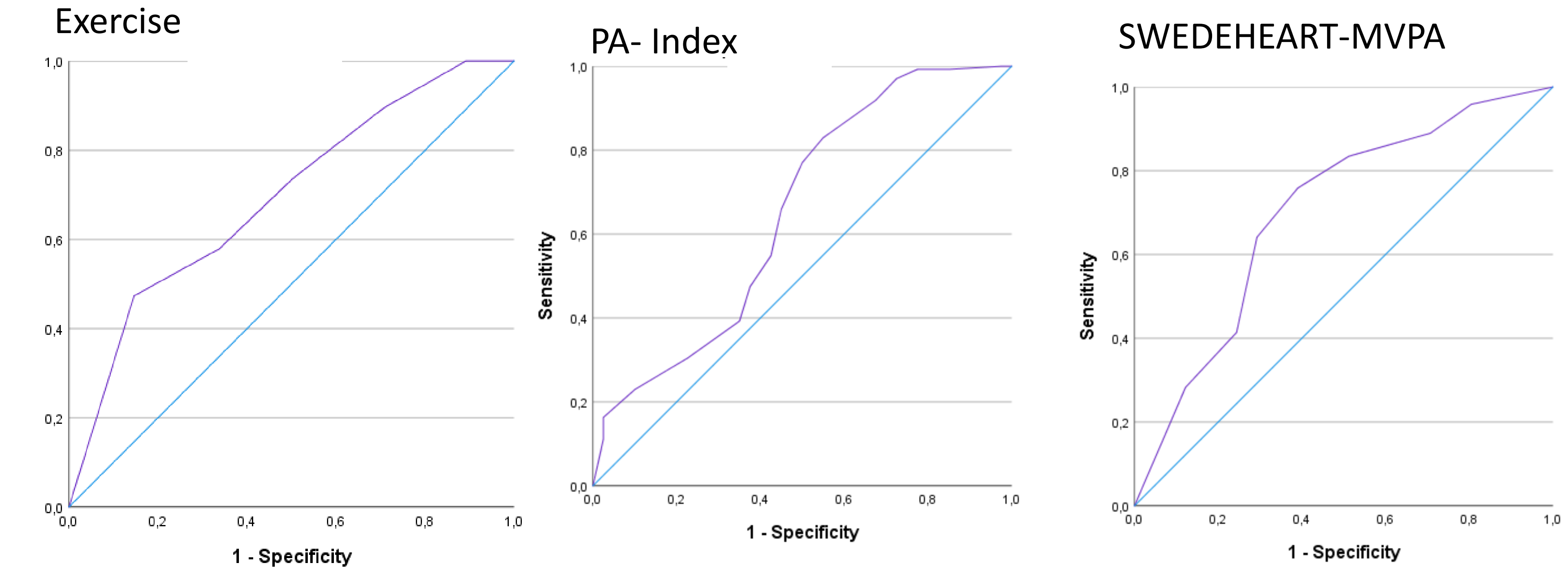

Appendix 2. Graphical results of the ROC analyses for the specificity and sensitivity of the PA and SED questions compared to accelerometer collected data
